# Hepatitis B virus genome mutations in precore and basal core promoter regions among HBeAg-negative chronic hepatitis B patients with high viral load in Indonesia

**DOI:** 10.1371/journal.pone.0357754

**Published:** 2026-09-15

**Authors:** Dewi Wulandari, Yan Mardian, Sunita, Kadek Aditya Darmayoga, Rindha Agustiani, Khoirunnisa, Youdiil Ophinni

**Affiliations:** 1 Department of Clinical Pathology, Faculty of Medicine, Universitas Indonesia, Cipto Mangunkusumo General Hospital, Jakarta, Indonesia; 2 The Hakubi Center for Advanced Research, Kyoto University, Kyoto, Japan; 3 Center for Southeast Asian Studies (CSEAS), Kyoto University, Kyoto, Japan; 4 Department of Host Defense, Immunology Frontier Research Center (IFReC), Osaka University, Osaka, Japan; 5 Division of Clinical Virology, Center for Infectious Diseases, Kobe University, Kobe, Japan; Children’s National Hospital, George Washington University, UNITED STATES OF AMERICA

## Abstract

Hepatitis B e antigen (HBeAg) is widely used as a marker for active HBV replication and serves as a surrogate for HBV DNA > 200,000 IU/mL to determine eligibility for tenofovir disoproxil fumarate (TDF) prophylaxis to prevent vertical transmission, according to WHO guidelines. However, some HBeAg-negative patients still harbor high viral loads. Mutations in the precore (PC) and basal core promoter (BCP) regions may reduce or abolish HBeAg expression without necessarily suppressing viral replication. Next-generation sequencing (NGS)-based characterization of these mutations remains limited in Indonesia. This study aimed to analyze the mutation prevalence in the BCP and PC regions associated with HBeAg negativity in Indonesian patients. We conducted a cross-sectional study of 32 chronic HBV treatment-naïve, unvaccinated patients with HBV DNA > 200,000 IU/mL (16 HBeAg-negative, 16 HBeAg-positive) at Cipto Mangunkusumo General Hospital. BCP and PC mutations were analyzed using NGS, classifying mutations as major (mutation frequency index [MFI] ≥20%) or minor (MFI 1– < 20%). Associations were analyzed using the Chi-square or Fisher’s exact test and p-values were adjusted using the Benjamini-Hochberg procedure. Among 29 major mutation sites, PC mutations A1846T/C and G1896A were more frequent in HBeAg-negative than HBeAg-positive patients (81.3% vs 6.3% and 75.0% vs 12.5%, respectively; all adjusted p = 0.019). Combined analysis showed higher mutation frequencies in HBeAg-negative patients (93.8%, 81.3%, and 62.5% for A1846T/C, G1896A, and G1899A, respectively; all adjusted p = 0.015). In conclusion, HBeAg-negative patients with high viral loads are strongly associated with PC mutations, particularly G1896A, A1846T/C, and G1899A. These exploratory findings provide regional NGS-based molecular evidence that established PC mutations may contribute to the coexistence of HBeAg negativity and continued high-level HBV replication in Indonesian patients. Larger studies incorporating broader virological and clinical comparison groups are required to determine the clinical significance of these findings.

## Introduction

Hepatitis B virus infection remains a global health problem, as World Health Organization (WHO) reported in Global Hepatitis Report 2024 estimating that 3.2% of the global population or 254 million individuals were chronically infected with HBV, with 1.2 million new cases and 1.1 million deaths reported annually [1]. More than half of those cases originated from the Asia Pacific region, including Indonesia. Based on Survei Kesehatan Indonesia *(Indonesia Health Survey)* 2023, the proportion of hepatitis B surface antigen (HBsAg) reactivity among the general population was 2.4% or about 7 million cases, and 0.1% among children under the age of 5 years [2]. However, the endemicity rate of HBV infection varies among provinces in Indonesia mainly due to geographic factors, health care coverage, and the quality of case reporting, with the highest rate reported being 4.7% in Papua Tengah Province, and the lowest was 0.5% in Daerah Istimewa Yogyakarta province [3]. Vertical transmission remains a significant public health concern, as the prevalence of HBsAg reactivity was reported in up to 1.5% of 3.5 million pregnant women tested. This may be a serious health burden, as more than 90% of vertically infected children during the perinatal period tend to develop chronic infection with higher risk of hepatic cirrhosis and hepatocellular carcinoma [4].

In 2023, the Indonesian Ministry of Health adopted the WHO 2020 guidelines for tenofovir disoproxil fumarate (TDF) prophylaxis for HBsAg-positive pregnant women, with eligibility criteria based on HBV DNA > 200,000 IU/mL (≥5.3 log_10_ IU/mL) or a positive hepatitis B e antigen (HBeAg) test[5,6]. The more recent WHO 2024 guidelines broadened the eligibility to include all HBsAg-positive pregnant women, particularly in settings with limited laboratory resources [7]. However, while these guidelines allow for broader TDF use, the cost-effectiveness of using HBeAg testing as an alternative to HBV DNA testing remains crucial in Indonesia. Not only is HBV DNA testing more expensive and reliant on PCR technology but providing TDF prophylaxis to all HBsAg-positive pregnant women, regardless of viral load or HBeAg status, could lead to unsustainable costs. In contrast, serum HBeAg testing is more affordable and widely available in many laboratories in Indonesia, making it a practical tool for identifying candidates for TDF prophylaxis, especially in resource-limited settings where both molecular testing and the supply of tenofovir are still limited.

Hepatitis B e antigen is a viral protein synthesized during the replication of the HBV and secreted by infected hepatocytes. As a result, HBeAg is commonly used as a marker of HBV replication and plays a crucial role in assessing the activity of chronic HBV infection. HBeAg is encoded by the PC/core gene within open reading frame (ORF) C of the HBV genome. This region is transcribed into PC mRNA, which is translated into the PC protein that undergoes post-translational processing to produce secreted HBeAg, while the core region encodes the hepatitis B core antigen (HBcAg). Mutations in the precore (PC) and basal core promoter (BCP) regions may disrupt HBeAg expression, resulting in an HBeAg-negative phenotype despite high levels of viral replication [8–10]. The G1896A substitution in the PC region introduces a premature stop codon that halts HBeAg synthesis. In addition, the A1762T and G1764A mutations in the BCP region decrease PC mRNA transcription, which in turn reduces HBeAg expression. Neither mutations interferes with viral replication, and therefore, may result in high HBV DNA without detectable HBeAg in the circulation [11,12].

In an unpublished preliminary institutional laboratory evaluation conducted in 2025 at Cipto Mangunkusumo General Hospital, a national tertiary referral hospital, approximately 34% of patients with high HBV DNA levels (>5 log10 IU/mL) had undetectable serum HBeAg. Serum HBeAg and quantitative HBV DNA were measured using the ARCHITECT HBeAg chemiluminescent microparticle immunoassay (Abbott) and the cobas HBV test on the cobas 6800/8800 Systems (Roche), respectively. This proportion is notably higher than the 27% reported in the United States and 29.3% in Canada [13,14]. These preliminary institutional observations have not been formally published and are therefore presented only as the clinical rationale underlying the present molecular investigation. Previous studies have shown that this discordant phenotype is most commonly associated with mutations in the BCP and PC regions, which reduce or abolish HBeAg expression without necessarily suppressing viral replication [8–10]. Other factors, such as analytical factors or other mutations leading to altered protein products undetected by conventional HBeAg assays, may also contribute.

Molecular studies in Indonesia have traditionally employed direct or Sanger sequencing to detect HBV mutations [15,16]. However, Sanger sequencing is limited in its ability to identify minor variants (<20%) or capture the full complexity of viral quasispecies. Quasispecies refer to a heterogeneous population of closely related but not identical viral genomes, which is characteristic of HBV due to its high replication rate and lack of proofreading capability in its polymerase. The viral population’s heterogeneity makes it difficult to detect variants present in low-abundance proportions. With the advent of next-generation sequencing (NGS), which enables large-scale, parallel analysis and generates hundreds to thousands of reads per sample, NGS has become a more powerful tool than Sanger sequencing in detecting both major and minor variants within the HBV quasispecies population, enabling a better understanding of the viral genome [17–19]. Despite its advantages, NGS-based molecular characterization of HBV remains relatively underexplored in Indonesia. To date, no study has specifically focused on evaluating mutations in the PC or BCP regions using NGS in cases where there is a discrepancy between HBeAg status and high HBV DNA levels in chronic hepatitis B patients. In this study, we aimed to comparatively identify and characterize mutations in the PC and BCP regions between HBeAg-negative patients with high circulating HBV DNA and a comparison group of HBeAg-positive patients with comparable viral loads, to elucidate the genetic determinants underlying this discordant phenotype.

## Materials and methods

### Sample

EDTA plasma samples were obtained from patients with chronic hepatitis B virus (HBV) infection who presented to Cipto Mangunkusumo General Hospital (RSCM) for routine diagnostic workup and clinical management between November 20, 2025, and January 30, 2026. All participants had no documented history of antiviral treatment or hepatitis B vaccination. HBeAg status was determined as part of routine serological testing using the ARCHITECT HBeAg chemiluminescent microparticle immunoassay (CMIA) (Abbott Diagnostics, Abbott Park, IL, USA; clinical sensitivity: 100.0%, specificity: ≥ 99.5%). Quantitative HBV DNA levels were measured using the cobas HBV test on the fully automated cobas 6800/8800 Systems (Roche Molecular Systems, Pleasanton, CA, USA). Only remnant EDTA plasma samples with high viral loads, defined as HBV DNA > 200,000 IU/mL (log_10_ 5.3 IU/mL), were utilized for subsequent molecular analysis. Samples with gross hemolysis, visible turbidity, lipemia, or severe icterus (bilirubin >20 mg/dL) were excluded.

The minimum sample size was calculated using a two-sided test for two independent proportions based on prior data (Utama et al., 2011) [15], with α = 0.05 and 80% power, yielding at least 9 subjects per group. In line with pilot study recommendations (≥12 per group) [20], and given the availability of eligible samples, all 16 eligible HBeAg-negative patients with HBV DNA levels >200,000 IU/mL were included. An equal number of HBeAg-positive patients meeting the same HBV DNA eligibility criterion (>200,000 IU/mL) were randomly selected from the same study period as the virological comparison group, resulting in a total sample size of 32. All samples were stored at −80 °C until analysis.

All laboratory procedures were performed at the Immunology and Biomolecular Laboratory, Integrated Laboratory Unit, Cipto Mangunkusumo General Hospital, and the Indonesia Medical Education and Research Institute (IMERI), Faculty of Medicine, Universitas Indonesia, Jakarta.

As a national tertiary teaching hospital (academic health care), RSCM implements an institutional general consent framework signed by patients at registration, which permits the secondary use of leftover or remnant clinical specimens and medical record data for scientific research, provided that subject anonymity and confidentiality are protected through complete de-identification. Given that this study exclusively utilized existing anonymized remnant materials collected during routine clinical evaluations—without requiring any additional diagnostic interventions, secondary procedures, or supplementary blood sampling—the requirement for study-specific individual informed consent was formally waived by the Ethics Committee. The study protocol was formally reviewed and approved by the Ethics Committee of the Faculty of Medicine, Universitas Indonesia – RSCM (No. KET-1406/UN2.F1/ETIK/PPM.00.02/2025). The approved research project was entitled in the original language as “Mutasi Genom Virus Hepatitis B pada Penderita Hepatitis B Kronis HBeAg Negatif dengan Viremia Tinggi di RSUPN Cipto Mangunkusumo” (translated into English as “Hepatitis B virus genome mutations in patients with HBeAg-negative chronic hepatitis B with high viral load at Cipto Mangunkusumo General Hospital”) and adhered strictly to the principles of the Declaration of Helsinki. Additional information regarding the ethical, cultural, and scientific considerations specific to inclusivity in global research is included in the Supporting Information (S1 Checklist).

### HBV DNA extraction and amplification

Viral DNA was isolated from EDTA plasma samples using the Nucleic acid extraction Kit (Zybio Inc. Chongqing, China) and semi-automatic nucleic acid extraction system (Nucleic Acid Isolation System Zybio EXM-3000) based on magnetic beads method. All processes were performed following the standard protocol of the manufacturer [21]. Following the isolation, DNA products were then amplified by PCR, followed by agarose gel electrophoresis to visualize the band of amplification products according to the size of DNA targets.

**Fig 1:**
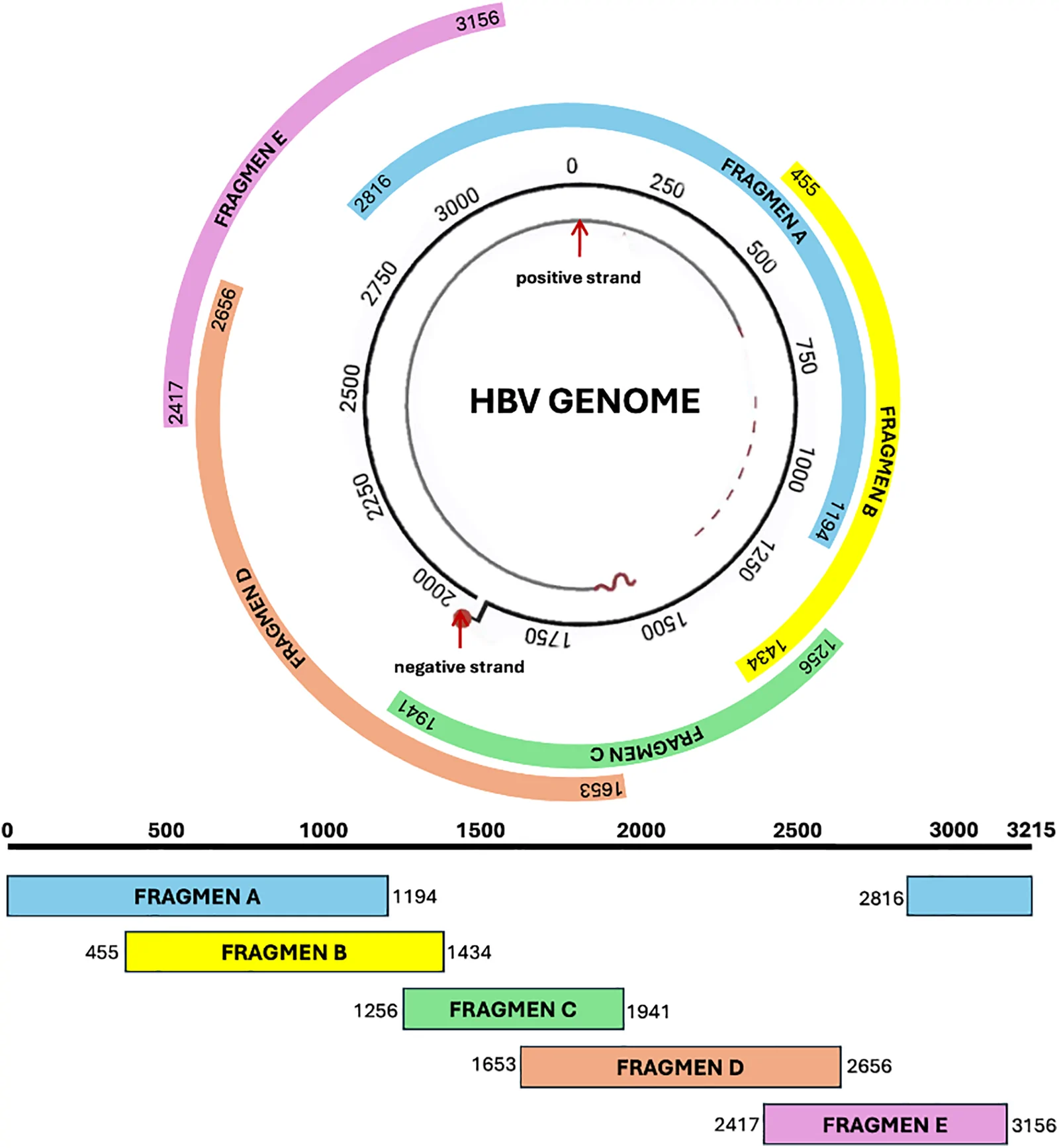
Overlapping target amplicons based on five primer sets.

Whole-genome amplification of HBV (+3,200 bp) was performed using 5 overlapping primer sets, as previously described by Thedja et al. (2011) (Fragment A to E, as shown in Fig 1), including PS8–1/HS6–2, S2-1/HB4R, HB5F/S013, PC1/HB9R, and HB10F/T734 [22]. Amplification was performed using Q5® Hot Start High-Fidelity 2X Master Mix (New England Biolabs, M0494) and Biorad CFX96 TM Real Time PCR system. PCR amplification consisted of an initial denaturation at 98 °C for 30 s, followed by 35 cycles of denaturation at 98 °C for 10 s, annealing at 62 °C for 20 s, and extension at 72 °C for 45 s, with a final extension at 72 °C for 120 s. Products were held at 4 °C until retrieval.

Amplicons were verified by electrophoresis in 1.5% agarose gel in TAE 1x buffer, and GelRed staining. A 1000 bp DNA ladder was used as band size marker. Electrophoresis was run at 100 V for 45 minutes and documented by gel documentation system. Expected amplicon sizes based on the primer design were 1593 bp for set primer 1 (Fragment A), 979 bp for set primer 2 (Fragment B), 685 bp for set primer 3 (Fragment C), 1003 bp for set primer 4 (Fragment D), and 739 bp for set primer 5 (Fragment E).

### Library preparation and sequencing

Library Preparation was conducted using Illumina Microbial Amplicon Prep (IMAP) Kit according to the standard protocol of the manufacturer, following PCR amplicon clean up using Illumina purification beads and standard IMAP-Flu library preparation workflow, which is applicable to custom amplicon panels beyond influenza [23,24]. The concentration of each library was measured using Qubit dsDNA HS Assay Kit after library indexing and clean up, with expected concentration between 10–100 ng/µL prior to pooling. The concentration of the pooled amplicon library (PAL) was re-measured with the Qubit dsDNA HS Assay Kit. The fragment sizes were analyzed using TapeStation, with an optimal fragment size range of 200–600 bp, yielding a unimodal peak. The final step in library preparation involved quantifying and normalizing the library to achieve a concentration of 4 nM.

The sequencing workflow was performed in accordance with the standard procedure of the manufacturer using the MiSeq Reagent Nano Kit v2 and Illumina MiSeq v2 Nano System. Quality control of the sequencing data was analyzed using Sequencing Analysis Viewer. The pre-defined acceptable criteria for sequencing quality metrics were a minimum raw reads Q30 > 80%, minimum total yield of 300 Mb, and minimum paired end reads of 2 million [25]. For each individual sample, the acceptable criteria for quality control included a minimum callable genome coverage of 90% and minimum median coverage of 200x [26]. All samples met these criteria and were further analyzed.

The sequencing data were further processed using the standardized, cloud-based Dragen Microbial Amplicon bioinformatics tool, integrated into the Illumina BaseSpace platform. This automated, non-adjustable pipeline applies highly stringent preprocessing quality control, conducting adapter trimming, off-target sequence removal, de-hosting, and bioinformatic de-duplication to eliminate duplicate reads prior to alignment. To systematically mitigate unidirectional strand bias, the pipeline executes consensus-building through the concurrent alignment of both forward and reverse paired-end reads against the reference genome before variant calling. The Mutation Frequency Index (MFI) value or Allele Fraction (AF) directly reflects the mutation frequency at particular nucleotide positions. Mutations with MFI between 1% and <20% were categorized as minor variants or quasispecies, while mutations with an MFI ≥ 20% were classified as major variants [27]. Variants detected near the 1% threshold were interpreted cautiously and labeled as strictly exploratory.

Genotyping was performed using phylogenetic algorithms based on the HBV genome database from GenBank, which includes all known HBV genotypes and subgenotypes. This analysis was conducted using MEGA version 7 software. Serotype determination was subsequently carried out according to the algorithms described by Yano et al. (2015) [28]. All HBV near-complete genome sequences generated in this study have been deposited in the NCBI GenBank database under BioProject accession PRJNA1469779, with the 32 corresponding BioSample accessions SAMN60375493–SAMN60375524 and GenBank nucleotide accessions PZ456435–PZ456466.

### Statistical analysis

Bioinformatic analysis was performed to determine mutation frequencies in the PC and BCP regions. Mutations in the BCP and PC regions were mapped against genotype-specific reference genomes, using HBV genotype B3 (subtype adw2; GenBank accession number AB713527.1) for genotype B samples and HBV genotype C1 (subtype adrq + ; GenBank accession number AB111946.1) for genotype C samples. Both major and minor variants were included in the analysis, covering the classical mutations G1896A and A1762T/G1764A, as well as other detected variants within these regions. Data were tabulated using Microsoft Excel, followed by statistical analysis conducted using SPSS version 20 software. Associations between the presence of major and minor mutations in the PC and BCP regions with HBeAg status were analyzed using the Chi-square test or Fisher’s exact test, as appropriate. Given the number of mutation sites tested, to account for multiple comparisons within each analysis, p-values were adjusted using the Benjamini-Hochberg procedure to control the false discovery rate at 5%. Corrections were applied separately for major variant analysis (29 comparisons), minor variant analysis (49 comparisons), and combined analysis (61 comparisons) as each analysis tests a biologically distinct hypothesis family (dominant viral population, low-abundance quasispecies, and overall mutation presence, respectively) for which per-family FDR control is appropriate. Effect sizes were reported as odds ratios (OR) with 95% confidence intervals (CI) calculated using the Woolf logarithmic method with a 0.5 continuity correction applied to cells containing zero counts. A sensitivity analysis using a normal approximation for two independent proportions was performed to determine the minimum detectable absolute risk difference at the achieved sample size. With 16 subjects per group, α = 0.05, and 80% power, the minimum detectable absolute risk difference was approximately 45 percentage points across the relevant range of baseline frequencies (10–50%). An adjusted p-value <0.05 was considered statistically significant.

## Results

### Quality Control Results of Viral DNA Amplicon

**Fig 2:**
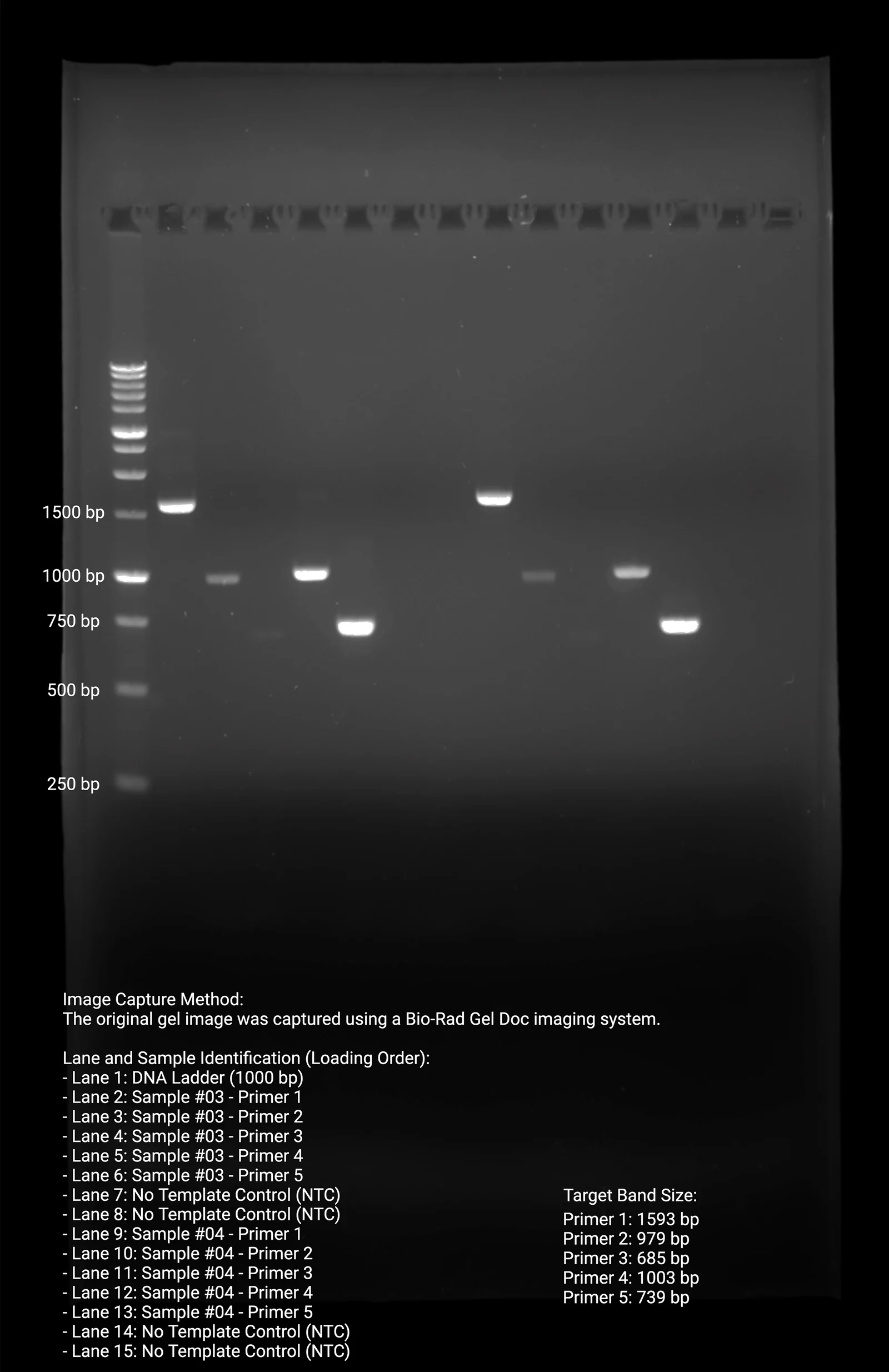
Agarose gel visualization of DNA amplicons electrophoresis of sample 03, sample 04, and NTC.

Gel electrophoresis confirmed that each PCR product yielded bands of the expected sizes for their respective target fragments. Fig 2 shows the representative results for Sample 03 and Sample 04 (the original, uncropped gel image is available as S1 Raw Image). The no-template controls (NTC) showed no amplification bands, confirming the absence of contamination

### Quality control results of DNA library

**Fig 3:**
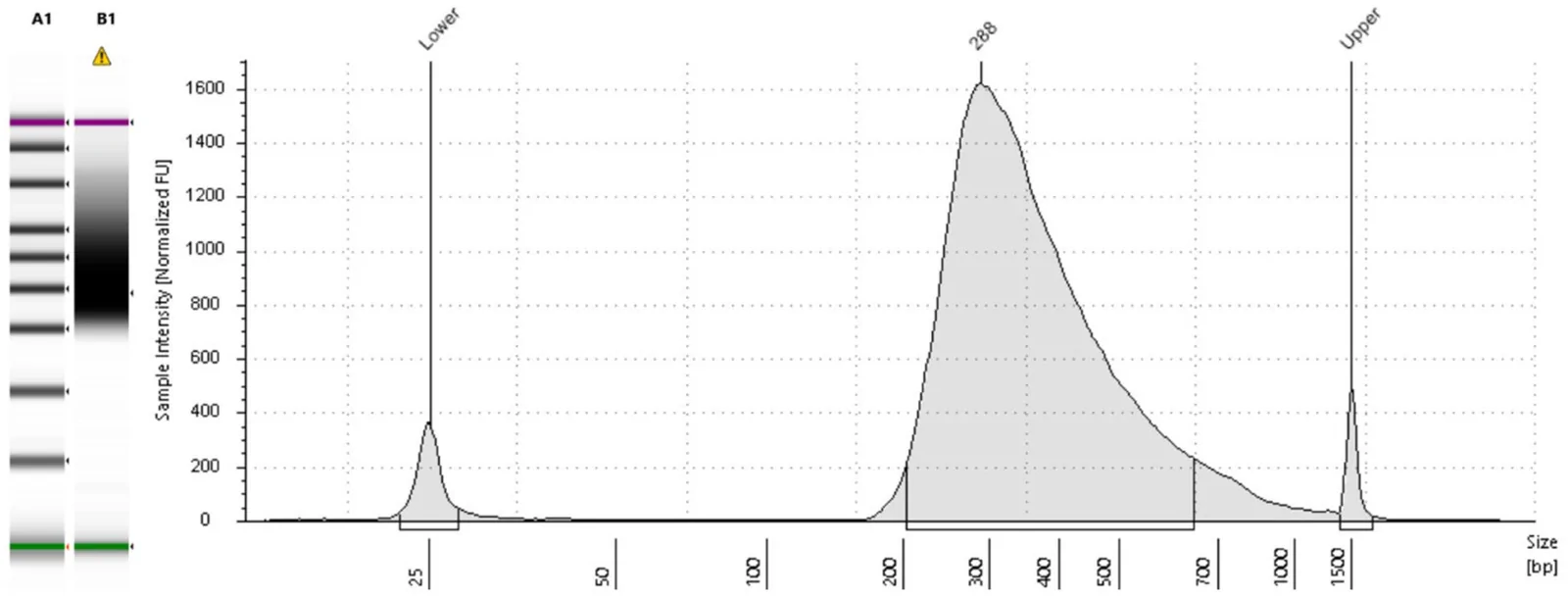
Fragment size distribution analysis of the library using TapeStation.

The measured library concentration across samples ranged from 36 to 56 ng/µL. Prior to pooling, the library of each sample was adjusted to a final concentration of 10 ng/µL. After pooling, the measured concentration was 0.843 ng/µL. Fragment analysis of the library pool revealed a dominant peak at 288 bp, a unimodal distribution, and no detectable adapter dimers in the 120–170 bp range as shown in Fig 3. With the concentration of 0.843 ng/µL and an average fragment size of 288 bp, the calculated library concentration was 4.4 nM.

### Sequencing metrics, genotyping, and serotyping analysis

Analysis of the sequencing metrics yielded a total output of 420 Mb, comprising 2.4 million paired-end reads, with 95.78% of bases exceeding Q30, meeting the performance specifications of the MiSeq Reagent Nano Kit v2. Median coverage ranged from 859 to 1749. The genome coverage (% of the genome callable) across all samples ranged from 99.78 to 100%.

Phylogenetic analysis revealed that 23 samples (71.88%) belonged to genotype B3, with 5 samples (15.63%) identified as genotype C. Serotype analysis showed that 23 samples were classified as Adw2 (71.88%). The complete data on genome coverage (% callable), median coverage, median genotype/subgenotype, and serotype are presented in **S1 Table**.

### Mutation analysis of the BCP and PC regions

**Fig 4:**
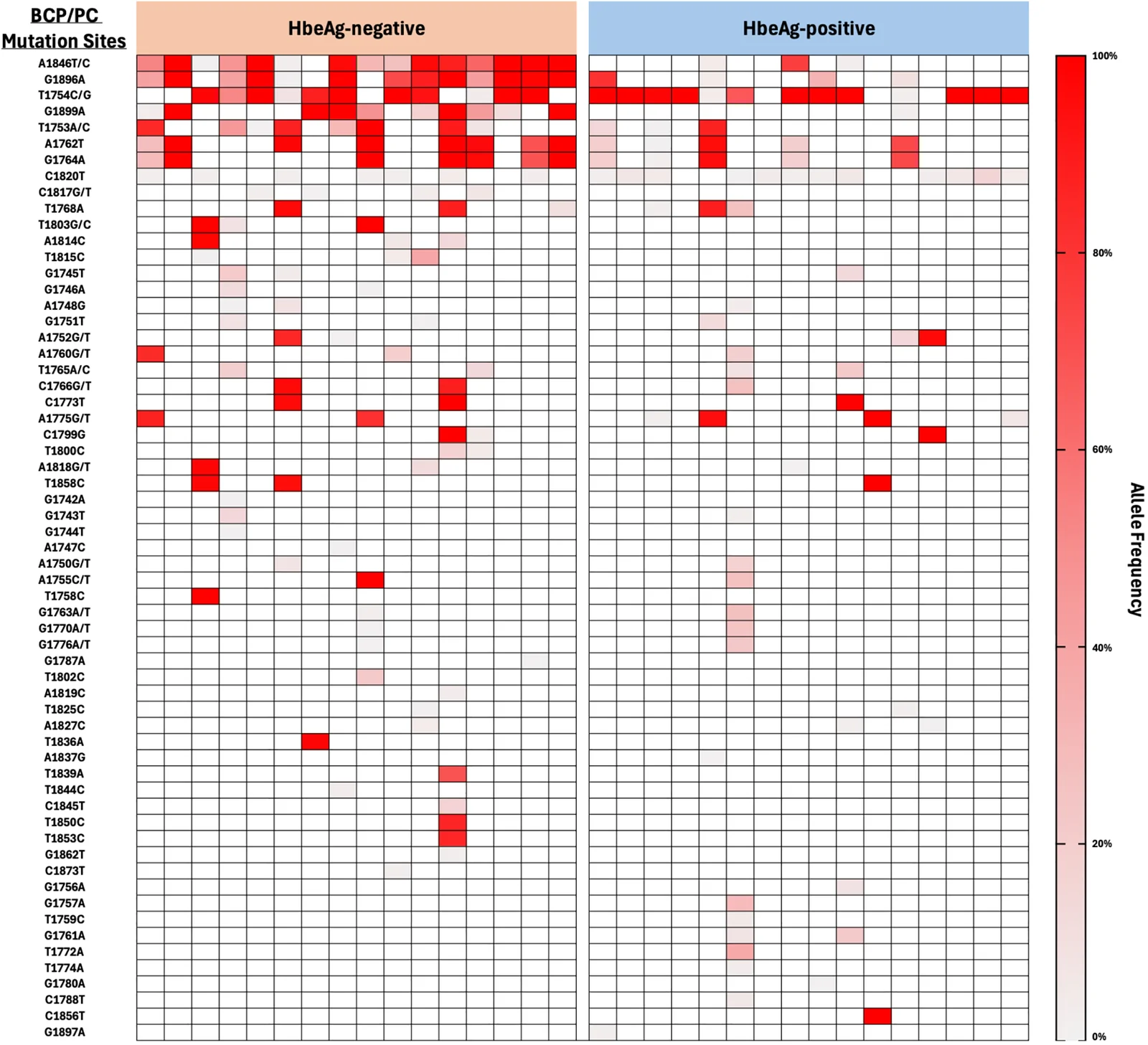
Heatmap showing the distribution of mutations across 61 differential sites between HBeAg-positive and HBeAg-negative patients.

The BCP region spans nt 1742–1849, whereas the PC region extends from nt 1814–1900, resulting in an overlapping segment at nt 1814–1849. A total of 61 differential mutation sites were identified between HBeAg-positive and HBeAg-negative patients. Their distribution and allele frequencies across samples are visualized in Fig 4 as a heatmap, demonstrating distinct mutation patterns between the two groups.

Major mutations were identified at 29 sites across the BCP, BCP/PC overlapping, and PC regions. Significant differences between HBeAg-positive and HBeAg-negative patients were primarily observed in mutations within the PC region. Among these, A1846T/C and G1896A were more frequent in the HBeAg-negative group than in the HBeAg-positive group (adjusted *p* = 0.019 and 0.019, respectively). Several BCP mutations, including T1753A/C, A1762T, and G1764A, also appeared more frequent in the HBeAg-negative group, but these differences did not reach statistical significance (all p > 0.05). The distribution and frequencies of all 29 major mutation sites in both groups are summarized in Table 1.

**Table 1:** Major mutation frequency analysis (MFI ≥ 20%) in the BCP and PC regions.

Major Mutations	Position/ Region	Number and Frequency of Major Mutations in the HBeAg Negative Group (N = 16)	Number and Frequency of Major Mutations in the HBeAg Positive Group (N = 16)	OR (95% CI)†	p-value	Adjusted p-value^***^
A1752G/T	BCP	1 (6.3%)	1 (6.3%)	1.00 (0.06–17.5)	1.000*	1.000
T1753A/C	BCP	6 (37.5%)	1 (6.3%)	9.00 (0.94–86.5)	0.083*	0.602
T1754C/G	BCP	9 (56.3%)	11 (68.8%)	0.58 (0.14–2.48)	0.467**	0.677
A1755C/T	BCP	1 (6.3%)	1 (6.3%)	1.00 (0.06–17.5)	1.000*	1.000
G1757A	BCP	0 (0%)	1 (6.3%)	0.31 (0.01–8.28)	0.234*	0.522
T1758C	BCP	1 (6.3%)	0 (0%)	3.19 (0.12–84.4)	0.234*	0.522
A1760G/T	BCP	1 (6.3%)	0 (0%)	3.19 (0.12–84.4)	0.234*	0.522
A1762T	BCP	7 (43.8%)	2 (12.5%)	5.44 (0.92–32.3)	0.113*	0.655
G1763A/T	BCP	0 (0%)	1 (6.3%)	0.31 (0.01–8.28)	0.234*	0.522
G1764A	BCP	6 (37.5%)	2 (12.5%)	4.20 (0.70–25.3)	0.220*	1.000
C1766G/T	BCP	2 (12.5%)	1 (6.3%)	2.14 (0.17–26.3)	1.000*	1.000
T1768A	BCP	2 (12.5%)	2 (12.5%)	1.00 (0.12–8.13)	1.000*	1.000
T1772A	BCP	0 (0%)	1 (6.3%)	0.31 (0.01–8.28)	0.234*	0.522
C1773T	BCP	2 (12.5%)	1 (6.3%)	2.14 (0.17–26.3)	1.000*	1.000
A1775G/T	BCP	2 (12.5%)	2 (12.5%)	1.00 (0.12–8.13)	1.000*	1.000
C1799G	BCP	1 (6.3%)	1 (6.3%)	1.00 (0.06–17.5)	1.000*	1.000
T1803G/C	BCP	2 (12.5%)	0 (0%)	5.69 (0.25–128.5)	0.484*	0.668
A1814C	BCP/PC	1 (6.3%)	0 (0%)	3.19 (0.12–84.4)	0.234*	0.522
T1815C	BCP/PC	1 (6.3%)	0 (0%)	3.19 (0.12–84.4)	0.234*	0.522
A1818G/T	BCP/PC	1 (6.3%)	0 (0%)	3.19 (0.12–84.4)	0.234*	0.522
T1836A	BCP/PC	1 (6.3%)	0 (0%)	3.19 (0.12–84.4)	0.234*	0.522
T1839A	BCP/PC	1 (6.3%)	0 (0%)	3.19 (0.12–84.4)	0.234*	0.522
A1846T/C	BCP/PC	13 (81.3%)	1 (6.3%)	65.0 (6.00–703.7)	<0.001^**^	**0.019** ^**+**^
T1850C	PC	1 (6.3%)	0 (0%)	3.19 (0.12–84.4)	0.234*	0.522
T1853C	PC	1 (6.3%)	0 (0%)	3.19 (0.12–84.4)	0.234*	0.522
C1856T	PC	0 (0%)	1 (6.3%)	0.31 (0.01–8.28)	0.234*	0.522
T1858C	PC	2 (12.5%)	1 (6.3%)	2.14 (0.17–26.3)	1.000*	1.000
G1896A	PC	12 (75.0%)	2 (12.5%)	21.0 (3.25–135.5)	<0.001^**^	**0.019** ^**+**^
G1899A	PC	7 (43.8%)	0 (0%)	26.1 (1.33–508.8)	0.007*	0.068

*Fisher’s exact test; **Chi-square test; ***p-values adjusted using the Benjamini-Hochberg procedure to control the false discovery rate at 5%; + *statistically significant (adjusted p < 0.05);* † *Odds ratios with 95% confidence intervals calculated using the Woolf logarithmic method; a 0.5 continuity correction was applied to cells containing zero counts. Wide CIs reflect the small per-group sample size; lower CI bounds of the two significant findings (A1846T/C, G1896A) and finding approaching significance (G1899A) all exceed 1, indicating consistent direction of association even at the conservative end of the interval.*

Minor mutations were identified at 49 sites across the BCP, BCP/PC overlapping, and PC regions, with no significant differences between HBeAg-negative and HBeAg-positive groups. Several sites previously identified as major mutations were also detected as minor variants, but their frequencies did not differ significantly between groups (Table 2).

**Table 2:** Minor mutation frequency analysis (MFI 1– < 20%) in the BCP and PC regions.

Minor Mutations	Position/ Region	Number and Frequency of Minor Mutations in the HBeAg Negative Group (N = 16)	Number and Frequency of Minor Mutations in the HBeAg Positive Group (N = 16)	OR (95% CI)†	P-value	Adjusted p-value***
G1742A	BCP	1 (6.3%)	0 (0%)	3.19 (0.12–84.4)	0.234*	0.566
G1743T	BCP	1 (6.3%)	1 (6.3%)	1.00 (0.06–17.5)	1.000*	0.725
G1744T	BCP	1 (6.3%)	0 (0%)	3.19 (0.12–84.4)	0.234*	0.566
G1745T	BCP	2 (12.5%)	1 (6.3%)	2.14 (0.17–26.3)	1.000*	0.725
G1746A	BCP	2 (12.5%)	0 (0%)	5.69 (0.25–128.5)	0.484*	0.573
A1747C	BCP	1 (6.3%)	0 (0%)	3.19 (0.12–84.4)	0.234*	0.566
A1748G	BCP	2 (12.5%)	1 (6.3%)	2.14 (0.17–26.3)	1.000*	0.725
A1750G/T	BCP	1 (6.3%)	1 (6.3%)	1.00 (0.06–17.5)	1.000*	0.725
G1751T	BCP	2 (12.5%)	1 (6.3%)	2.14 (0.17–26.3)	1.000*	0.725
A1752G/T	BCP	1 (6.3%)	1 (6.3%)	1.00 (0.06–17.5)	1.000*	0.725
T1753A/C	BCP	2 (12.5%)	2 (12.5%)	1.00 (0.12–8.13)	1.000*	0.725
T1754C/G	BCP	2 (12.5%)	2 (12.5%)	1.00 (0.12–8.13)	1.000*	0.725
G1756A	BCP	0 (0%)	1 (6.3%)	0.31 (0.01–8.28)	0.234*	0.566
T1759C	BCP	0 (0%)	1 (6.3%)	0.31 (0.01–8.28)	0.234*	0.566
A1760G/T	BCP	1 (6.3%)	1 (6.3%)	1.00 (0.06–17.5)	1.000*	0.725
G1761A	BCP	0 (0%)	2 (12.5%)	0.18 (0.01–3.97)	0.484*	0.573
A1762T	BCP	1 (6.3%)	3 (18.8%)	0.29 (0.03–3.13)	0.600*	0.600
G1763A/T	BCP	1 (6.3%)	0 (0%)	3.19 (0.12–84.4)	0.234*	0.566
G1764A	BCP	1 (6.3%)	3 (18.8%)	0.29 (0.03–3.13)	0.600*	0.600
T1765A/C	BCP	2 (12.5%)	2 (12.5%)	1.00 (0.12–8.13)	1.000*	0.725
T1768A	BCP	1 (6.3%)	1 (6.3%)	1.00 (0.06–17.5)	1.000*	0.725
G1770A/T	BCP	1 (6.3%)	1 (6.3%)	1.00 (0.06–17.5)	1.000*	0.725
T1774A	BCP	0 (0%)	1 (6.3%)	0.31 (0.01–8.28)	0.234*	0.566
A1775G/T	BCP	0 (0%)	2 (12.5%)	0.18 (0.01–3.97)	0.484*	0.573
G1776A/T	BCP	1 (6.3%)	1 (6.3%)	1.00 (0.06–17.5)	1.000*	0.725
G1780A	BCP	0 (0%)	1 (6.3%)	0.31 (0.01–8.28)	0.234*	0.566
G1787A	BCP	1 (6.3%)	0 (0%)	3.19 (0.12–84.4)	0.234*	0.566
C1788T	BCP	0 (0%)	1 (6.3%)	0.31 (0.01–8.28)	0.234*	0.566
C1799G	BCP	1 (6.3%)	0 (0%)	3.19 (0.12–84.4)	0.234*	0.566
T1800C	BCP	2 (12.5%)	0 (0%)	5.69 (0.25–128.5)	0.484*	0.573
T1802C	BCP	1 (6.3%)	0 (0%)	3.19 (0.12–84.4)	0.234*	0.566
T1803G/C	BCP	1 (6.3%)	0 (0%)	3.19 (0.12–84.4)	0.234*	0.566
A1814C	BCP/PC	2 (12.5%)	0 (0%)	5.69 (0.25–128.5)	0.484*	0.573
T1815C	BCP/PC	2 (12.5%)	0 (0%)	5.69 (0.25–128.5)	0.484*	0.573
C1817G/T	BCP/PC	4 (25.0%)	0 (0%)	11.9 (0.58–241.7)	0.101*	1.000
A1818G/T	BCP/PC	1 (6.3%)	1 (6.3%)	1.00 (0.06–17.5)	1.000*	0.725
A1819C	BCP/PC	1 (6.3%)	0 (0%)	3.19 (0.12–84.4)	0.234*	0.566
C1820T	BCP/PC	7 (43.8%)	12 (75.0%)	0.26 (0.06–1.16)	0.071**	1.000
T1825C	BCP/PC	1 (6.3%)	1 (6.3%)	1.00 (0.06–17.5)	1.000*	0.725
A1827C	BCP/PC	1 (6.3%)	2 (12.5%)	0.47 (0.04–5.73)	1.000*	0.725
A1837G	BCP/PC	1 (6.3%)	1 (6.3%)	1.00 (0.06–17.5)	1.000*	0.725
T1844C	BCP/PC	1 (6.3%)	0 (0%)	3.19 (0.12–84.4)	0.234*	0.566
C1845T	BCP/PC	1 (6.3%)	0 (0%)	3.19 (0.12–84.4)	0.234*	0.566
A1846T/C	BCP/PC	2 (12.5%)	2 (12.5%)	1.00 (0.12–8.13)	1.000*	0.725
G1862T	PC	1 (6.3%)	0 (0%)	3.19 (0.12–84.4)	0.234*	0.566
C1873T	PC	1 (6.3%)	0 (0%)	3.19 (0.12–84.4)	0.234*	0.566
G1896A	PC	1 (6.3%)	2 (12.5%)	0.47 (0.04–5.73)	1.000*	0.725
G1897A	PC	0 (0%)	1 (6.3%)	0.31 (0.01–8.28)	0.234*	0.566
G1899A	PC	3 (18.8%)	1 (6.3%)	3.46 (0.32–37.5)	0.600*	0.600

*Fisher’s exact test; **Chi-square test; ***p-values adjusted using the Benjamini-Hochberg procedure to control the false discovery rate at 5; † *Odds ratios with 95% confidence intervals calculated using the Woolf logarithmic method; a 0.5 continuity correction was applied to cells containing zero counts.*

When major and minor variants were analyzed together, a total of 61 mutation sites were identified across the BCP, BCP/PC overlapping, and PC regions. The combined analysis remained consistent with the findings from the major mutation analysis. Mutations at nucleotide positions 1846, 1896, and 1899 within the PC region were significantly more frequent in the HBeAg-negative group. The inclusion of minor variants increased the mutation detection frequency in HBeAg-negative samples to 93.8% for A1846T/C, 81.3% for G1896A, and 62.5% for G1899A, all of which remained statistically significant (all adjusted p = 0.015). Mutations within the BCP region continued to show no significant differences between HBeAg-negative and HBeAg-positive groups. The distribution of all 61 mutation sites identified across both regions is summarized in Table 3.

**Table 3:** Combined analysis of major and minor mutations in the BCP and PC regions.

Major + Minor Mutations	Position/ Region	Number and Frequency of Major + Minor Mutations in the HBeAg Negative Group (N = 16)	Number and Frequency of Major + Minor Mutations in the HBeAg Positive Group (N = 16)	OR (95% CI)†	P-value	Adjusted p-value^***^
G1742A	BCP	1 (6.3%)	0 (0%)	3.19 (0.12–84.4)	0.234*	0.316
G1743T	BCP	1 (6.3%)	1 (6.3%)	1.00 (0.06–17.5)	1.000*	0.569
G1744T	BCP	1 (6.3%)	0 (0%)	3.19 (0.12–84.4)	0.234*	0.316
G1745T	BCP	2 (12.5%)	1 (6.3%)	2.14 (0.17–26.3)	1.000*	0.569
G1746A	BCP	2 (12.5%)	0 (0%)	5.69 (0.25–128.5)	0.484*	0.379
A1747C	BCP	1 (6.3%)	0 (0%)	3.19 (0.12–84.4)	0.234*	0.316
A1748G	BCP	2 (12.5%)	1 (6.3%)	2.14 (0.17–26.3)	1.000*	0.569
A1750G/T	BCP	1 (6.3%)	1 (6.3%)	1.00 (0.06–17.5)	1.000*	0.569
G1751T	BCP	2 (12.5%)	1 (6.3%)	2.14 (0.17–26.3)	1.000*	0.569
A1752G/T	BCP	2 (12.5%)	2 (12.5%)	1.00 (0.12–8.13)	1.000*	0.569
T1753A/C	BCP	8 (50.0%)	3 (18.8%)	4.33 (0.88–21.3)	0.063**	0.457
T1754C/G	BCP	11 (68.8%)	13 (81.3%)	0.51 (0.10–2.62)	0.685*	0.497
A1755C/T	BCP	1 (6.3%)	1 (6.3%)	1.00 (0.06–17.5)	1.000*	0.569
G1756A	BCP	0 (0%)	1 (6.3%)	0.31 (0.01–8.28)	0.234*	0.316
G1757A	BCP	0 (0%)	1 (6.3%)	0.31 (0.01–8.28)	0.234*	0.316
T1758C	BCP	1 (6.3%)	0 (0%)	3.19 (0.12–84.4)	0.234*	0.316
T1759C	BCP	0 (0%)	1 (6.3%)	0.31 (0.01–8.28)	0.234*	0.316
A1760G/T	BCP	2 (12.5%)	1 (6.3%)	2.14 (0.17–26.3)	1.000*	0.569
G1761A	BCP	0 (0%)	2 (12.5%)	0.18 (0.01–3.97)	0.484*	0.379
A1762T	BCP	8 (50.0%)	5 (31.3%)	2.20 (0.52–9.30)	0.280**	0.239
G1763A/T	BCP	1 (6.3%)	1 (6.3%)	1.00 (0.06–17.5)	1.000*	0.569
G1764A	BCP	7 (43.8%)	5 (31.3%)	1.71 (0.40–7.27)	0.465**	0.385
T1765A/C	BCP	2 (12.5%)	2 (12.5%)	1.00 (0.12–8.13)	1.000*	0.569
C1766G/T	BCP	2 (12.5%)	1 (6.3%)	2.14 (0.17–26.3)	1.000*	0.569
T1768A	BCP	3 (18.8%)	3 (18.8%)	1.00 (0.17–5.90)	1.000*	0.569
G1770A/T	BCP	1 (6.3%)	1 (6.3%)	1.00 (0.06–17.5)	1.000*	0.569
T1772A	BCP	0 (0%)	1 (6.3%)	0.31 (0.01–8.28)	0.234*	0.316
C1773T	BCP	2 (12.5%)	1 (6.3%)	2.14 (0.17–26.3)	1.000*	0.569
T1774A	BCP	0 (0%)	1 (6.3%)	0.31 (0.01–8.28)	0.234*	0.316
A1775G/T	BCP	2 (12.5%)	4 (25.0%)	0.43 (0.07–2.76)	0.654*	0.486
G1776A/T	BCP	1 (6.3%)	1 (6.3%)	1.00 (0.06–17.5)	1.000*	0.569
G1780A	BCP	0 (0%)	1 (6.3%)	0.31 (0.01–8.28)	0.234*	0.316
G1787A	BCP	1 (6.3%)	0 (0%)	3.19 (0.12–84.4)	0.234*	0.316
C1788T	BCP	0 (0%)	1 (6.3%)	0.31 (0.01–8.28)	0.234*	0.316
C1799G	BCP	2 (12.5%)	1 (6.3%)	2.14 (0.17–26.3)	1.000*	0.569
T1800C	BCP	2 (12.5%)	0 (0%)	5.69 (0.25–128.5)	0.484*	0.379
T1802C	BCP	1 (6.3%)	0 (0%)	3.19 (0.12–84.4)	0.234*	0.316
T1803G/C	BCP	3 (18.8%)	0 (0%)	8.56 (0.41–180.5)	0.226*	0.819
A1814C	BCP/PC	3 (18.8%)	0 (0%)	8.56 (0.41–180.5)	0.226*	0.819
T1815C	BCP/PC	3 (18.8%)	0 (0%)	8.56 (0.41–180.5)	0.226*	0.819
C1817G/T	BCP/PC	4 (25.0%)	0 (0%)	11.9 (0.58–241.7)	0.101*	0.488
A1818G/T	BCP/PC	2 (12.5%)	1 (6.3%)	2.14 (0.17–26.3)	1.000*	0.569
A1819C	BCP/PC	1 (6.3%)	0 (0%)	3.19 (0.12–84.4)	0.234*	0.316
C1820T	BCP/PC	7 (43.8%)	12 (75.0%)	0.26 (0.06–1.16)	0.071**	0.412
T1825C	BCP/PC	1 (6.3%)	1 (6.3%)	1.00 (0.06–17.5)	1.000*	0.569
A1827C	BCP/PC	1 (6.3%)	2 (12.5%)	0.47 (0.04–5.73)	1.000*	0.569
T1836A	BCP/PC	1 (6.3%)	0 (0%)	3.19 (0.12–84.4)	0.234*	0.316
A1837G	BCP/PC	1 (6.3%)	1 (6.3%)	1.00 (0.06–17.5)	1.000*	0.569
T1839A	BCP/PC	1 (6.3%)	0 (0%)	3.19 (0.12–84.4)	0.234*	0.316
T1844C	BCP/PC	1 (6.3%)	0 (0%)	3.19 (0.12–84.4)	0.234*	0.316
C1845T	BCP/PC	1 (6.3%)	0 (0%)	3.19 (0.12–84.4)	0.234*	0.316
A1846T/C	BCP/PC	15 (93.8%)	3 (18.8%)	65.0 (6.00–703.7)	<0.001^**^	**0.015** ^**+**^
T1850C	PC	1 (6.3%)	0 (0%)	3.19 (0.12–84.4)	0.234*	0.316
T1853C	PC	1 (6.3%)	0 (0%)	3.19 (0.12–84.4)	0.234*	0.316
C1856T	PC	0 (0%)	1 (6.3%)	0.31 (0.01–8.28)	0.234*	0.316
T1858C	PC	2 (12.5%)	1 (6.3%)	2.14 (0.17–26.3)	1.000*	0.569
G1862T	PC	1 (6.3%)	0 (0%)	3.19 (0.12–84.4)	0.234*	0.316
C1873T	PC	1 (6.3%)	0 (0%)	3.19 (0.12–84.4)	0.234*	0.316
G1896A	PC	13 (81.3%)	4 (25.0%)	13.0 (2.40–70.5)	<0.001^**^	**0.015** ^**+**^
G1897A	PC	0 (0%)	1 (6.3%)	0.31 (0.01–8.28)	0.234*	0.316
G1899A	PC	10 (62.5%)	1 (6.3%)	25.0 (2.60–240.3)	0.001^**^	**0.015** ^**+**^

*Fisher’s exact test; **Chi-square test, ***p-values adjusted using the Benjamini-Hochberg procedure to control the false discovery rate at 5%, † *Odds ratios with 95% confidence intervals calculated using the Woolf logarithmic method; a 0.5 continuity correction was applied to cells containing zero counts. Wide CIs reflect the small per-group sample size; lower CI bounds for the three significant findings (A1846T/C, G1896A, G1899A) all exceed 1, indicating consistent direction of association even at the conservative end of the interval.*

## Discussion

Chronic hepatitis B virus infection remains a major global public health challenge, including in Indonesia. Although the national hepatitis B vaccination program for infants has substantially reduced HBV prevalence, considerable regional variation persists, and vertical transmission remains an important source of new infections in several provinces. Perinatal HBV transmission is particularly concerning because infants infected at birth have a high risk of developing chronic infection, which may later progress to cirrhosis or hepatocellular carcinoma at relatively young ages [4]. To prevent mother-to-child transmission, the World Health Organization recommends tenofovir prophylaxis during pregnancy for women with high viral replication, defined by plasma HBV DNA levels exceeding 200,000 IU/mL or the presence of HBeAg [5]. In many regions of Indonesia, however, access to quantitative HBV DNA testing remains limited, and HBeAg is frequently used as a practical surrogate marker to guide prophylactic therapy. This approach requires careful interpretation because a substantial proportion of patients with high viral loads may present with an HBeAg-negative phenotype.

Mutations in the BCP and PC regions of the HBV genome are strongly associated with HBeAg negativity, regardless of the viral replication status [29]. The BCP region regulates transcriptional initiation of PC messenger RNA and pregenomic RNA (pgRNA), both of which are essential for viral replication. The PC region encodes the HBeAg precursor and contains regulatory elements involved in pgRNA synthesis and viral genome packaging [12]. Mutations within these regions may therefore disrupt HBeAg production without necessarily suppressing viral replication, leading to an HBeAg-negative phenotype despite high plasma HBV DNA levels [11,12]. The present study thus aimed to investigate the prevalence of mutations in the BCP and PC regions that may contribute to reduced HBeAg expression in patients with high viral loads.

Classic mutations involving BCP nucleotides 1762/1764 and PC nucleotides 1896/1899 are among the best characterized and have been consistently associated with reduced HBeAg expression [29]. HBeAg has important immunomodulatory functions that facilitate viral persistence during chronic HBV infection, although it is not required for viral replication [30]. Accordingly, these mutations may reduce or abolish HBeAg production without impairing viral replication, resulting in an HBeAg-negative phenotype despite persistent viremia [11,31]. These variants are reported most frequently in genotype B and C infections and are particularly prevalent in Asian populations.

Most previous studies have relied on conventional sequencing, which primarily detects dominant viral populations and provides limited insight into the full spectrum of HBV quasispecies [13,15,16]. Next-generation sequencing enables more comprehensive detection of both major and minor variants within the viral population [27]. In this study, only treatment-naïve and unvaccinated patients were included to minimize treatment- or vaccine-induced immune pressure that might influence the distribution of BCP and PC mutations [32]. The inclusion of treatment- and vaccine-naïve subjects in this study suggests that these observations may reflect the phenotype of the virus circulating within the population.

Within this context, the principal contribution of the present study lies in its regional and methodological perspective rather than in identifying a previously unknown association between PC mutations and HBeAg negativity. Although the relationship between classical PC mutations, particularly G1896A, and loss of HBeAg expression has been well established, NGS-based characterization of PC and BCP variants remains limited in Indonesia. By applying high-depth sequencing to an Indonesian cohort dominated by HBV subgenotype B3, this study provides regional molecular data while enabling simultaneous assessment of both dominant and low-frequency variants that may not be detected by conventional consensus sequencing. We found that PC mutations were significantly associated with the HBeAg-negative phenotype, whereas BCP mutations were present in both groups without significant differences. This pattern is consistent with previous reports showing that PC mutations, particularly around nucleotides 1896 and 1899, are more directly linked to loss of HBeAg expression while allowing continued viral replication. Conversely, BCP mutations are distributed across different phases of chronic HBV infection and do not consistently distinguish HBeAg-negative from HBeAg-positive patients [11,15,29]. Taken together, these findings support the view that, in this cohort, PC mutations represent the more immediate molecular basis for the absence of detectable HBeAg despite ongoing viral replication.

The reliability of the mutation analysis was supported by consistent amplification and high-quality sequencing performance. Specific PCR amplification confirmed accurate target enrichment, while sequencing metrics met the expected specifications of the MiSeq Nano v2 platform. Genome coverage exceeded 99% across samples, with sequencing depth far above the commonly recommended ~200 × threshold for reliable viral variant detection. Such depth is essential for resolving HBV quasispecies, enabling the identification of both dominant mutations and low-frequency variants within the viral population [25,26,33]. Collectively, these metrics indicate that the sequencing dataset was sufficiently robust to support reliable downstream interpretation of PC and BCP mutations in this cohort.

The molecular context of these mutations should also be considered in relation to the circulating HBV genetic background. In this cohort, most isolates belonged to genotype B3 with serotype adw2, a distribution that reflects the dominant HBV strains reported in Indonesia and several Southeast Asian populations. Previous studies have shown that the genomic background of HBV genotypes can influence the emergence and stability of mutations within the PC and BCP regions, particularly those affecting HBeAg expression [34,35]. The predominance of genotype B in this cohort may therefore provide a favorable genomic context for the emergence of PC variants associated with HBeAg loss [29]. This distribution accurately reflects the local molecular epidemiology, rendering these findings highly relevant and impactful for Indonesian clinical strategies. Given this regional context, genotype-adjusted analyses were not performed. Instead, reference sequences specific to genotypes B and C were utilized from the study’s inception to align with the most prevalent strains in Asia.

Among the mutations identified, G1896A emerged as one of the most prominent variants, occurring at a markedly higher frequency in the HBeAg-negative group. This nonsense mutation in the HBV PC region involves a G-to-A substitution that converts codon 28 from TGG (tryptophan) to the premature stop codon TAG. This alteration terminates translation of the PC protein and abolishing HBeAg synthesis while permitting continued viral replication [11,12]. This study also found G1896A mutation significantly occurred in HBeAg-negative samples, consistent with previous studies. Importantly, the predominance of G1896A observed in our HBeAg-negative high-HBV-DNA cohort is consistent with this well-established molecular mechanism, which has been widely reported in HBV genotypes B and C [11,36].

In these genotypes, the presence of Thymine (T) at nucleotide 1858 allows the G1896A substitution to form a stable A1896–T1858 base pair within the ε stem-loop structure. This configuration preserves the integrity of the pregenomic RNA encapsidation signal required for viral replication. Consequently, although HBeAg synthesis is abolished due to the premature stop codon introduced by the G1896A mutation, the structural stability of the encapsidation signal permits continued viral replication. This mechanism maintains high circulating HBV DNA levels [11,36]. Conversely, in non-B/C strains such as genotype A, nucleotide position 1858 is typically occupied by a Cytosine (C). A G1896A mutation would thus introduce a disruptive C-A mismatch that destabilizes the encapsidation loop, theoretically compromising viral survival [12,13]. While a small subset of genotype C isolates (*n* = 5) in our cohort demonstrated qualitative mutational trends similar to genotype B, their limited number precluded separate statistical sub-analysis. To definitively validate these genotype-specific structural dynamics across diverse geographical regions in Indonesia, future large-scale, multicenter, and population-based evaluations are warranted.

Because the present study included only patients with HBV DNA levels >200,000 IU/mL, it was not designed to determine whether these mutations are associated specifically with persistent high viral replication or with HBeAg loss alone. Nevertheless, within this predefined high-HBV-DNA population, the observed mutation profile is consistent with the established molecular mechanism underlying HBeAg loss with preserved viral replication. However, future studies incorporating HBeAg-negative patients with both low and high HBV DNA levels are needed. Such studies would allow direct comparison of mutation frequencies between these groups and help distinguish mutations associated primarily with HBeAg loss from those associated with persistent high viral replication.

In addition, this study also identified mutations A1846T/C and G1899A in the PC region among HBeAg-negative patients. These findings are consistent with a previous study by Chen et al., which reported increased frequencies of mutations at nucleotide positions 1846, 1896, and 1899 in HBeAg-negative HBV infections [37]. The G1899A mutation involves a guanine-to-adenine substitution at nucleotide 1899 that results in a glycine-to-aspartic acid change at amino acid residue 29 of the PC protein. This variant has been proposed to stabilize the ε lower-stem structure required for pgRNA encapsidation, thereby supporting continued viral replication despite reduced HBeAg expression [38].

Nonetheless, A1846T is considered as silent nucleotide substitution that does not alter the amino acid sequence of the PC protein. Although this mutation has been linked to HBeAg-negative infection and more advanced liver disease, its direct mechanistic contribution to impaired HBeAg expression remains incompletely understood [37,39]. Because functional assays evaluating viral translation efficiency or pathogenicity were outside the scope of this preliminary study, this statistical correlation should not be overinterpreted as direct biological causality. Instead, given its close genomic proximity to known functional variants within the precore region, the high prevalence of A1846T/C may be attributed to linkage disequilibrium or a genetic co-selection effect with adjacent mutations. This co-selection likely occurs with adjacent mutations, such as G1896A and G1899A, which also dominate the HBeAg-negative high viral load group. Consequently, this finding remains hypothesis-generating. Further in vitro functional validation is required to delineate whether A1846T/C exerts any independent phenotypic effects or serves purely as a genetic marker for nearby functional clusters.

Within the BCP region, this study observed mutation T1753A/C, A1762T, and G1764A in both groups without significant differences, although their frequency tended to be higher in HBeAg-negative samples. This observation is consistent with previous reports indicating that BCP mutations may occur across different immune phases of chronic HBV infection and are therefore not restricted to the HBeAg-negative state [40]. Among these variants, the A1762T/G1764A double mutation is the best-characterized promoter alteration. It reduces transcription of PC mRNA, leading to decreased HBeAg production without completely abolishing antigen expression [11,12]. In an *in vivo* analysis of HBV transcription, Laras et al. reported that PC mRNA synthesis was suppressed by the A1762T/G1764A mutation, demonstrating that the principal effect of this mutation occurs at the transcriptional level [41]. In addition, longitudinal studies have shown that these mutations may be detected in both HBeAg-positive and anti-HBe-positive phases of infection, as noted by Fang et al., indicating that their presence alone does not define the HBeAg-negative phenotype [40].

Mutations at nucleotide 1753 (T1753V; C/A/G) represent additional regulatory changes within the BCP region that may further modify promoter activity. Previous studies have suggested that substitutions at this position can influence viral transcription and may act synergistically with A1762T/G1764A in modulating viral replication and HBeAg expression [11,12]. In addition, T1753 mutations have been associated with advanced liver disease, including cirrhosis and hepatocellular carcinoma, and have been reported as potential risk markers for HCC in several populations [12]. Together, these findings suggest that BCP alterations may contribute to reduced HBeAg expression but are less directly linked to complete HBeAg loss than canonical PC stop-codon mutations such as G1896A.

An important aim of this study was to explore whether mutations in the PC or BCP regions could lead to structural changes in the HBeAg protein, potentially evading detection by commercial immunoassays. Antigenic escape has been proposed as a possible explanation for cases where patients with high viral loads test negative for HBeAg. This phenomenon is well-documented for HBsAg, where mutations such as G145R and other S-gene immune escape mutants can impair antigenicity and interfere with assay binding [42]. However, in the present study, the mutations identified in this cohort were primarily linked to reduced or abolished HBeAg production, rather than structural changes in the protein that would prevent assay recognition. Specifically, the G1896A mutation leads to premature termination of the HBeAg protein, abolishing its expression. In contrast, BCP mutations predominantly affect the transcriptional regulation of PC mRNA, reducing HBeAg synthesis at the transcriptional level. None of the mutations observed in this study correspond to known HBeAg antigenic escape variants that would alter epitope structure recognized by commercial assays. Therefore, these findings suggest that the discordance between HBeAg negativity and high viral load in this cohort is primarily explained by reduced HBeAg production associated with established PC mutations rather than by structural changes affecting HBeAg detection. This molecular insight provides biological context for interpreting discordant HBeAg and HBV DNA results in clinical laboratory practice.

This study highlights the significance of NGS in detecting both major mutations (MFI ≥ 20%) and minor variants (MFI 1– < 20%) within the HBV quasispecies [27]. The inclusion of minor variants in our analysis –particularly A1846T/C, G1896A, and G1899A– substantially increased mutation detection in the HBeAg-negative group. This result underscores the advantage of deep sequencing approaches for identifying low-frequency variants that may be missed by conventional sequencing [17]. Such low-frequency variants may represent viral subpopulations that warrant further investigation. Because the present study was cross-sectional and did not evaluate haplotype structure or longitudinal viral evolution, their temporal emergence, persistence, or expansion cannot be determined. Accordingly, the present findings should be interpreted as mutation-level observations rather than a comprehensive analysis of intra-host HBV quasispecies dynamics. Nevertheless, the intrinsic per-base error rate of the Illumina MiSeq is estimated at 0.1–1%. Therefore, validating minor variants near the 1% detection boundary through replicate sequencing or digital PCR would strengthen the reliability of minor variant identification in future studies.

Although the present work was designed strictly as molecular investigation, the findings may have implications for laboratory diagnostic interpretation. The predominance of established PC mutations among HBeAg-negative patients with persistently high HBV DNA levels provides a molecular explanation for the coexistence of HBeAg negativity and ongoing viral replication in a subset of patients with chronic HBV infection. These findings demonstrate that HBeAg-negative status may coexist with persistently high HBV DNA levels and therefore should not automatically be interpreted as evidence of low viral replication when accurate assessment of viral activity is clinically important. In such situations, quantitative HBV DNA measurement remains essential whenever available. However, the present study did not evaluate pregnancy outcomes, mother-to-child transmission, TDF prophylaxis eligibility, or the diagnostic performance of HBeAg relative to quantitative HBV DNA testing. Correlations between these molecular findings and clinical outcomes—including pregnancy outcomes, vertical transmission, liver disease severity, and other clinicopathological parameters—were beyond the current scope and warrant future investigation.

To investigate the molecular characteristics associated with this virological phenotype, we enrolled a general cohort of chronic HBV patients with high viral loads. This approach was considered appropriate because the fundamental molecular pathogenesis, replication dynamics, and mutational behavior of HBV are generally considered to be similar in pregnant and non-pregnant individuals. Gestation primarily modulates maternal host immune responses rather than intrinsic viral characteristics [43]. Given that pregnant individuals constitute a vulnerable research population, this study was designed as an initial molecular investigation in a well-defined cohort that can inform future investigations in pregnancy-specific populations. This stepwise approach enables the molecular basis of the phenotype to be established before its clinical relevance is evaluated in pregnancy-specific populations.

While this study provides valuable insights, several limitations should be acknowledged. First, the sample size was determined a priori from a two-proportion power calculation (Utama et al., 2009) yielding a minimum of 9 per group. This cohort was expanded to 16 per group in line with pilot-study recommendations and to maximize use of all eligible HBeAg-negative high-viral-load patients available during the study window. Sensitivity analysis indicates that this sample size is powered to detect absolute risk differences of approximately 45 percentage points or greater. Smaller true differences in mutation frequency between the HBeAg-negative and -positive groups may therefore be present but undetected (type II error), particularly within the minor-variant analyses. Notably, low-frequency minor variants sitting near the 1% threshold were identified in only three samples within this cohort (one HBeAg-positive and two HBeAg-negative isolates). Although these data were processed using DRAGEN’s automated bioinformatic de-duplication and paired-end consensus filters, these specific borderline threshold variations are interpreted cautiously and regarded as exploratory rather than definitive pending larger cohort validations. The principal findings — G1896A, A1846T/C, and G1899A more frequent in HBeAg-negative patients — involve risk differences of 50–75 percentage points and odds ratios of 12–65. These metrics substantially exceed the minimum detectable effect. Furthermore, they are biologically plausible given the well-established mechanism by which precore-region mutations abolish HBeAg synthesis (G1896A premature stop codon) or reduce precore mRNA transcription. Nevertheless, given the limited sample size, these molecular findings as a whole must be interpreted as exploratory, and further validation in larger, multicenter cohorts is required to confirm these mutational trends.

Second, the analysis focused on specific regions of the HBV genome, leaving other genomic regions unexplored where additional mutations could influence antigen expression or immune escape. In addition, while next-generation sequencing allowed detection of both major and minor variants, this analysis focused on individual mutations and did not include formal measures of intra-host quasispecies diversity, such as nucleotide diversity, haplotype reconstruction, or linkage analysis. This was a choice due to data constraints. Since libraries were amplicon-based and the MiSeq per-base error rate nears the 1% threshold for defining minor variants, diversity metrics at these levels could be confounded by amplification and sequencing noise. A comprehensive quasispecies-diversity assessment would need replicate sequencing or orthogonal validation, which we suggest as a future research. Functional studies were also not performed; therefore, the biological significance of several mutations with high prevalence in this study, such as A1846T/C, remains uncertain and requires further investigation.

Finally, the use of stored plasma samples originating from a single national tertiary referral hospital may introduce referral bias. Hence, these findings cannot be extrapolated to represent the general chronic HBV population across the country, nor are they generalizable to non-B/C HBV genotypes. Furthermore, the present study was designed as a molecular investigation rather than a clinicopathological cohort study. Consequently, detailed clinical variables, including liver biochemistry (ALT/AST), fibrosis stage, pregnancy outcomes, and longitudinal follow-up data, were not incorporated into the present analysis. This limits the direct clinical interpretation and translation of the observed molecular associations. Nevertheless, this study serves as an important pilot evaluation and provides regional NGS-based molecular data on PC and BCP mutations in Indonesian patients with high HBV DNA levels. Larger multicenter studies integrating molecular and clinical data across diverse geographical regions in Indonesia are warranted to validate these findings and determine their broader clinical significance.

## Conclusion

In conclusion, this study demonstrated that HBeAg-negative patients with high viral loads correlate with PC mutations, particularly G1896A, A1846T/C, and G1899A. These findings provide regional NGS-based data on PC and BCP mutation patterns and reinforce the established molecular basis for the coexistence of HBeAg negativity and persistent viral replication. This molecular evidence further highlights that HBeAg-negative status does not necessarily indicate low viral replication, providing a biological explanation for the observed discordance between HBeAg status and HBV DNA levels. This study also highlights the utility of next-generation sequencing in characterizing both major mutations and low-frequency minor variants within the viral quasispecies, offering a deeper resolution of the HBV genomic landscape in Indonesia. Larger, multicenter studies incorporating broader virological and clinical populations are required to determine the clinical significance of these findings.

## Supporting information

S1 Table Sequencing Results and Determination of HBV Genotype and Serotype for All Samples. (DOCX)

S1 Checklist Inclusivity in global research questionnaire. (DOCX)

S1 Raw Image Original Uncropped and Unadjusted Gel Image. (PDF)
