# Supplementary material for: Hepatitis B virus genome mutations in precore and basal core promoter regions among HBeAg-negative chronic hepatitis B patients with high viral load in Indonesia
Source: PLoS One. 2026 Sep 15;21(9):e0357754. doi: 10.1371/journal.pone.0357754 (PMC13577486; doi:10.1371/journal.pone.0357754)
Supplement: Supplementary file 1 [file pone.0357754.s003.pdf]

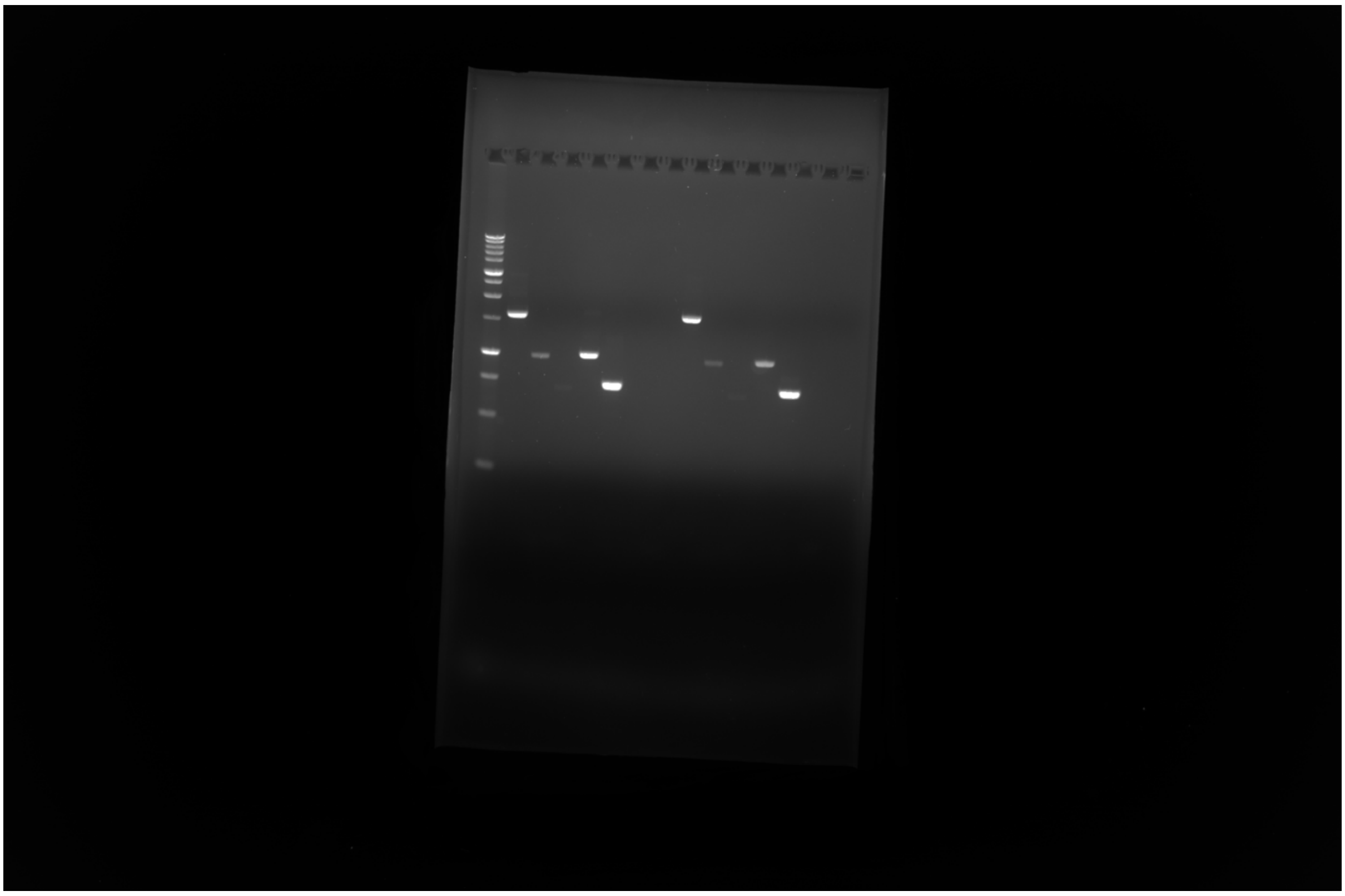

S1_raw_image: Original Uncropped and Unadjusted Gel Image

- **Figure Reference:** This original, uncropped, and unadjusted gel image corresponds to Figure 2 in the main manuscript.
- **Image Capture Method:** The image was captured using a Bio-Rad Gel Doc imaging system.
- **Lane and Sample Identification (Loading Order):**
    - **Lane 1:** DNA Ladder (1000 bp)
    - **Lane 2:** Sample #03 - Primer 1
    - **Lane 3:** Sample #03 - Primer 2
    - **Lane 4:** Sample #03 - Primer 3
    - **Lane 5:** Sample #03 - Primer 4
    - **Lane 6:** Sample #03 - Primer 5
    - **Lane 7:** No Template Control (NTC)
    - **Lane 8:** No Template Control (NTC)
    - **Lane 9:** Sample #04 - Primer 1
    - **Lane 10:** Sample #04 - Primer 2
    - **Lane 11:** Sample #04 - Primer 3
    - **Lane 12:** Sample #04 - Primer 4
    - **Lane 13:** Sample #04 - Primer 5
    - **Lane 14:** No Template Control (NTC)
    - **Lane 15:** No Template Control (NTC)
