# Supplementary material for: Hepatitis B virus genome mutations in precore and basal core promoter regions among HBeAg-negative chronic hepatitis B patients with high viral load in Indonesia
Source: PLoS One. 2026 Sep 15;21(9):e0357754. doi: 10.1371/journal.pone.0357754 (PMC13577486; doi:10.1371/journal.pone.0357754)
Supplement: Supplementary file 2 [file pone.0357754.s001.docx]

**Supplementary Table 1.** Sequencing Results and Determination of HBV Genotype and Serotype for All Samples

| **Sample Codes** | **% Callable** | **Median Coverage** | **Genotype/Subgenotype** | **Serotype** |
| --- | --- | --- | --- | --- |
| **01** | 100 | 1106 | B3 | Adw2 |
| **02** | 100 | 1431 | B3 | Adw2 |
| **03** | 100 | 1749 | B3 | Adw2 |
| **04** | 100 | 1373 | B3 | Adw2 |
| **05** | 100 | 1139 | C | Ayr |
| **06** | 100 | 1352 | B3 | Adw2 |
| **07** | 99.78 | 1349 | C | Adrq+ |
| **08** | 100 | 1281 | B3 | Adw3 |
| **09** | 100 | 1293 | B3 | Adw3 |
| **10** | 100 | 1347 | B3 | Adw2 |
| **11** | 100 | 1503 | C1 | Adrq+ |
| **12** | 100 | 1287 | B3 | Adw2 |
| **13** | 100 | 1502 | B2 | Adw2 |
| **14** | 100 | 1580 | B3 | Adw2 |
| **15** | 100 | 1440 | B3 | Adw2 |
| **16** | 100 | 1512 | B3 | Adw2 |
| **17** | 99,88 | 859 | C | Adw2 |
| **18** | 100 | 1312 | B3 | Adw4 |
| **19** | 100 | 1304 | B8 | Adw2 |
| **20** | 100 | 1397 | B3 | Adw2 |
| **21** | 100 | 1276 | B3 | Adw2 |
| **22** | 100 | 1382 | B8 | Ayw1 |
| **23** | 100 | 1151 | B3 | Adw2 |
| **24** | 100 | 1220 | B3 | Adw2 |
| **25** | 100 | 1486 | C | Adrq+ |
| **26** | 100 | 1485 | B3 | Adw2 |
| **27** | 100 | 1372 | B3 | Adw2 |
| **28** | 100 | 1262 | B2 | Ayw1 |
| **29** | 100 | 943 | B3 | Adw2 |
| **30** | 100 | 1508 | B3 | Adw2 |
| **31** | 100 | 1070 | B3 | Adw2 |
| **32** | 99,91 | 1467 | B3 | Adw2 |
