# Supplementary material for: Hepatitis B virus genome mutations in precore and basal core promoter regions among HBeAg-negative chronic hepatitis B patients with high viral load in Indonesia
Source: PLoS One. 2026 Sep 15;21(9):e0357754. doi: 10.1371/journal.pone.0357754 (PMC13577486; doi:10.1371/journal.pone.0357754)
Supplement: Supplementary file 3 [file pone.0357754.s002.docx]

Inclusivity in global research

PLOS’ policy on inclusivity in global research aims to improve transparency in the reporting of research performed outside of researchers’ own country or community and ensures that PLOS publications reporting global research adhere to high standards for research ethics and authorship. Authors of relevant research articles may be asked to complete the questionnaire below, which outlines ethical, cultural, and scientific considerations specific to inclusivity in global research. This questionnaire may be requested when researchers have travelled to a different country to conduct research, if research uses samples collected in another country, research with Indigenous populations or their lands, or if research is on cultural artefacts. Researchers travelling to another country solely to use laboratory equipment will not normally be required to complete the questionnaire. However, the questionnaire can be requested at the journal’s discretion for any submission – if you have been requested to complete this questionnaire by the PLOS journal you submitted to, please do so.

Please complete the questionnaire below and include this as a Supporting Information file with your manuscript. Note that if your paper is accepted for publication, this checklist will be published with your article in the supporting information files. Please ensure that you reference the checklist in the main body of your manuscript. We suggest adding a subsection ‘Inclusivity in global research’ to your Methods section and adding the following sentence: “Additional information regarding the ethical, cultural, and scientific considerations specific to inclusivity in global research is included in the Supporting Information (SX Checklist)”

The questions have been designed to be applicable to a wide range of study types, and there are subsections for both human subjects research and non-human subjects research. If any of the questions are not relevant to your research please mark them as “N/A” as appropriate.

**Ethical considerations, permits and authorship**

*This section is applicable to all research types.*

Provide details as to who granted permissions and/or consent for the study to take place in the Methods section of your manuscript. This should include the names of **all** ethics boards, governmental organizations, community leaders or other bodies that provided approval for the study. If individuals provided approval refer to these people by their role or title but do not list their name(s).

Reported on page number: #07

Formal institutional ethical approval for this study was reviewed and officially granted by the Ethics Committee of the Faculty of Medicine, Universitas Indonesia – Cipto Mangunkusumo General Hospital (RSCM) under reference number **No. KET-1406/UN2.F1/ETIK/PPM.00.02/2025**. This detail is explicitly provided in the "Sample" subsection under the Materials and Methods section of the manuscript.

If there were any deviations from the study protocol after approval was obtained please provide details of these changes in the Methods section of your manuscript.

Reported on page number: N/A

There were no deviations, modifications, or amendments made to the study protocol after receiving formal approval from the institutional Ethics Committee.

Did this study involve local collaborators that are residents of the country where the research was conducted or members of the community studied? If you do not have any authors from said communities, please provide an explanation for this below.

Yes. This study is an investigator-initiated research entirely conceived, managed, and executed by a 100% native Indonesian research team. The lead clinical investigators and laboratory personnel (D.W., S., Y.M., K.A.D., R.A., K.) are full-time residents of Indonesia and permanently affiliated with the host institution, Cipto Mangunkusumo General Hospital (RSCM) / Faculty of Medicine, Universitas Indonesia. The last author, Dr. Youdiil Ophinni (Y.O.), is also an Indonesian national who is currently based at academic institutions in Japan. He served as an equal collaborative partner providing specialized molecular and bioinformatic analysis. This study represents a completely symmetric and equitable partnership among Indonesian scientists, ensuring absolute local leadership, data ownership, and scientific execution within the host country.

Everyone listed as an author should meet PLOS’ criteria for authorship and all individuals who meet these criteria should be included in the author byline, rather than the acknowledgements. For further information please see the journal’s Authorship Policy.

Yes. All investigators and laboratory personnel listed in the author byline substantially contributed to the study's conceptualization, sample processing, library preparation, sequencing data analysis, and manuscript preparation. Every individual meets the standard PLOS criteria for authorship and is fully included in the author byline. No eligible contributors have been omitted or relegated to the acknowledgments.

**Human subjects research (e.g. health research, medical research, cross-cultural psychology)**

Did you obtain written informed consent from a representative of the local community or region before the research took place? How did you establish who speaks for the community? Details of written informed consent obtained from study participants should be reported separately in the Methods section of your manuscript.

How did members of the local community provide input on the aims of the research investigation, its methodology, and its anticipated outcome(s)?

As an investigator-initiated study designed and conducted entirely by local Indonesian clinical pathologists at RSCM, input from the local medical community was inherent to the project. The study aims were established directly from clinical gaps identified in daily local patient management, specifically the high viral loads observed in HBeAg-negative individuals. The methodology was tailored to leverage routine institutional diagnostic platforms for initial sample screening and characterization, integrated with existing internal molecular sequencing infrastructure to ensure a practical and sustainable workflow. Furthermore, the anticipated outcomes directly address national public health strategies to prevent vertical HBV transmission, providing actionable data for local clinicians and healthcare policymakers.

This study utilized existing, fully anonymized remnant (leftover) EDTA plasma samples retrieved from the clinical laboratory repository of a national tertiary teaching hospital or academic health care in Indonesia (RSCM). Upon initial hospital presentation, all patients routinely sign an institutional general consent framework that explicitly includes permission for the secondary use of residual clinical specimens for scientific research, provided that complete de-identification and anonymity are maintained. Because this study strictly utilized these anonymized residual materials without direct patient interaction or any additional supplementary clinical intervention, the requirement for study-specific individual informed consent was formally reviewed and waived by the Ethics Committee.

When engaging with the local community, how did you ensure that the informed consent documents and other materials could be understood by local stakeholders?

Because this study was a laboratory-based evaluation utilizing existing, fully anonymized residual clinical specimens under a formal waiver of individual informed consent granted by the Ethics Committee, no study-specific recruitment materials, consent forms, or community engagement documents were newly developed or administered for this project. The overarching institutional general consent framework is a standardized, routine administrative component of the hospital's central admission process. During the ethical protocol review, the Ethics Committee evaluated this institutional framework and determined it to be fully sufficient to protect participant autonomy and privacy, thereby granting the formal study-specific consent waiver.

Will the findings of the research be made available in an understandable format to stakeholders in the community where the study was conducted (e.g. via a presentation, summary report, copies of publications, etc.)? Please provide details of how this will be achieved.

Yes. The research findings will be actively disseminated locally through scientific presentations at national Indonesian clinical pathology and gastrohepatoenterology medical conferences, internal hospital seminars at Cipto Mangunkusumo General Hospital, and through the open-access publication of this manuscript in PLOS ONE, making the data freely available to local clinicians, medical students, and public health policy stakeholders in Indonesia.

**Non-human subjects research using specimens/ animals collected as part of the study, or those housed in archival collections. Examples include archaeology, paleontology, botany and zoology.**

Did the permission you obtained from a local authority to perform the study include an agreement on access to outputs and benefit sharing? This may include procedures to enable fair distribution of the benefits and resources arising from the research performed. Please include any details of Prior Informed Consent and Benefit Sharing Agreements obtained. These may be required by field-specific regulations, for example the Convention on Biological Diversity (CBD) and the associated Nagoya Protocol.

N/A

If the material used in your study was imported, please A) provide the year it was imported and B) indicate whether permits were obtained to import/export the materials used, C) provide details of any permits obtained. If this information is not available, please indicate this.

N/A

If you used archival specimens, please state how the material used in your study was acquired by the institute it is held in and provide details of any permits obtained for the original excavations/ sample collection. If this information is not available, please indicate this.

N/A

How was the potential cultural significance of the materials collected in your study to local communities considered in your research design? Were Indigenous peoples and/or local researchers and institutions involved with archaeological excavations / collection of specimens? If so, please provide a description of their involvement.

N/A

If your manuscript includes photographs of human remains please indicate whether authors obtained permission from descendants or affiliated cultural communities to do so.

N/A
